# The value of urodynamic tools to guide patient selection in sacral neuromodulation

**DOI:** 10.1007/s00345-015-1479-6

**Published:** 2015-02-14

**Authors:** Jamie Drossaerts, Kevin Rademakers, Gommert van Koeveringe, Philip Van Kerrebroeck

**Affiliations:** Department of Urology, Maastricht University Medical Centre (MUMC+), The Netherlands and School of Mental Health and Neuroscience (MHeNS), Maastricht University, P. Debyelaan 25, POB 5800, 6202 AZ Maastricht, The Netherlands

**Keywords:** Sacral neuromodulation, Refractory overactive bladder, Non-obstructive urinary retention, Urodynamics, Conventional urodynamic study, Ambulatory monitoring

## Abstract

**Purpose:**

The aim of this study is to explore whether urodynamics, with the addition of ambulatory urodynamic study (ambulatory-UDS), will be able to better predict and assess sacral neuromodulation (SNM) treatment outcome. Selection of patients is a critical element in achieving optimal outcome in SNM. Quantitative and qualitative results of urodynamic tests are used to justify surgical therapy and to evaluate treatment for lower urinary tract dysfunction. Therefore, these tests should be representative and subsequently offer a correct prognosis.

**Methods:**

Between December 2002 until May 2013 selected patients with lower urinary tract symptoms (storage and/or voiding dysfunction) were included in an ambulatory urodynamic measurement database. From this database, the total subgroup of patients that underwent a sacral neuromodulation test evaluation was selected.

**Results:**

A total of 98 patients were included. Success rate of SNM in patients with storage dysfunction was around 70 %, according to either conventional-UDS or ambulatory-UDS diagnosis. Based on conventional-UDS, success rate of SNM in patients with hypocontractility was 67 % and in acontractile patients 35 %. According to ambulatory-UDS diagnosis, success rates were 32 and 17 %, respectively.

**Conclusions:**

This study shows that conventional-UDS overestimates the amount of patients diagnosed with hypocontractile or acontractile bladder. Patients with reduced contractility on ambulatory-UDS have a lower chance of SNM success. Hence, ambulatory-UDS allows us to select patients with a real acontractile bladder and predict SNM failure. In patients with storage dysfunction, additional ambulatory-UDS does not seem to contribute in predicting SNM outcome.

## Introduction

Since the 1990s, sacral neuromodulation (SNM) is recommended as a secondary treatment option if conservative treatments either fail or lead to adverse events in patients with overactive bladder syndrome (OAB) or non-obstructive urinary retention (NOR). OAB syndrome is defined by the International Continence Society (ICS) as urgency, frequency and/or nocturia with or without urgency incontinence [1]. NOR has a multifactorial aetiology and can be caused by detrusor underactivity (DU), detrusor-bladder neck dyssynergia, detrusor-external sphincter dyssynergia, dysfunctional voiding and non-relaxing urethral sphincter obstruction. Treatment success of these storage and voiding dysfunctions is evaluated in terms of improvement in micturition diaries, subjective patient evaluation, quality of life scores and symptom score questionnaires.

Conventional urodynamic studies (conventional-UDS) use retrograde bladder filling via a catheter to monitor storage and voiding in a patient. Ambulatory urodynamic studies (ambulatory-UDS) aim to evaluate storage and voiding during natural filling of the bladder [2]. Since the development of ambulatory-UDS in the 1990s [2, 3], many studies have been performed to assess the utility of ambulatory-UDS in clinical practice. Ambulatory urodynamic studies were standardised in 2000 [4], but have yet to be clinically validated. It can be expected that the ambulatory-UDS technique is more sensitive in recording pressure/flow changes than conventional urodynamic measurements, which are currently the gold standard in urodynamics. However, ambulatory-UDS may also be more sensitive to artifacts and could be more prone to record or elicit non-physiological detrusor behaviour [5]. Others mention that the rapid filling in conventional-UDS could elicit artifacts and/or mask (other) artifacts that do manifest themselves during ambulatory-UDS [6]. These and other factors make validation a complex task. Nonetheless, it remains a necessary step in the clinical application of ambulatory-UDS. To correctly validate ambulatory-UDS, urodynamic results of conventional-UDS and ambulatory-UDS should be compared with each other on a large scale and both should be related to treatment outcome in different treatment modalities. One of these treatment modalities is SNM. In most studies with SNM treatment, success is defined as a reduction in one or more micturition symptoms of ≥50 %, compared to baseline, determined by comparing voiding diaries. However, also changes in urodynamic parameters during SNM have been reported by several research groups. In patients with NOR treated with SNM, a significant correlation was found between improvement in symptoms and changed conventional-UDS recording [7–9]. Several researchers found a similar significant correlation in OAB patients treated with SNM [10–12].

In this article, outcomes of conventional-UDS and ambulatory-UDS in patients evaluated with SNM are reviewed with a focus on the treatment outcome. The aim of this study is to explore to what extent a urodynamic diagnosis, with the addition of ambulatory-UDS, will be able to predict sacral neuromodulation treatment outcome even before SNM test evaluation is performed.

## Patients and methods

Selected patients underwent, besides a conventional-UDS, an additional ambulatory-UDS before starting (if any) treatment. Reasons for ambulatory-UDS were as follows: (1) low-quality conventional-UDS (2) assumed OAB syndrome without detrusor overactivity on conventional-UDS (3) incontinence with unclear primary origin (4) suspected bladder acontractility and (5) enuresis nocturna. All patients in this study underwent conventional and ambulatory urodynamic monitoring before treatment evaluation with sacral neuromodulation between December 2002 and April 2013. Clinical informed consent was obtained from all patients for the additional ambulatory-UDS. All urodynamic measurements were performed with the use of Medical Measurements Systems (MMS B.V., Enschede, The Netherlands) equipment and according to the standardisation report of the ICS [4, 13]. Any phasic contraction during the filling phase with rise and fall in detrusor pressure is diagnostic of detrusor overactivity (DO). The ICS definition does not specify a minimum change in detrusor pressure, although waves of an amplitude <5 cm H_2_O are difficult to detect. Bladder acontractility was defined as a filling and voiding phase without detrusor pressure rise. Hypocontractility was identified as a low detrusor pressure (<10 cm H_2_O) during the voiding phase, relative to the degree of obstruction, not resulting in (efficient) micturition. In the case of a conventional-UDS, the hypocontractility definition is elaborated with a peak flow rate (Qmax) of <10 mL/s, voided volume should be >100 mL and residual urine volume >150 mL [14]. For ambulatory-UDS standardised limits have not yet been established. The quality of all ambulatory-UDS recordings was probed, and the full results were interpreted by a staff member specialised in urodynamics and a resident experienced in judging urodynamic measurements. SNM treatment success is defined as a reduction in one or more micturition symptoms of ≥50 %, compared to baseline, determined by comparing voiding diaries.

### Statistical analysis

Descriptive and comparative statistics were calculated with the use of SPSS, IBM corporation, version 20. Median and 25–75 percentile interquartile range (IQR) were stated. Nominal association variables and measurement of agreement between conventional-UDS and ambulatory-UDS were calculated. Logistic regression analysis was performed to compare success rate by conventional and ambulatory urodynamic diagnoses.

## Results

A total of 98 patients underwent conventional-UDS and ambulatory-UDS before the neuromodulation test. The median age of included patients was 54 years (IQR 45–62), and the group consisted of 67 women and 31 men. Reasons for conducting a conventional-UDS were OAB (*n* = 25: wet, *n* = 3: dry), mixed urinary incontinence (*n* = 11) and voiding problems (*n* = 59). In 44 patients, the ambulatory-UDS was conducted after an inconclusive or unrepresentative conventional-UDS, in 40 patients because of alleged acontractility or hypocontractility, and in 14 patients because of OAB symptoms without DO on conventional-UDS. The median (IQR) duration of an ambulatory-UDS was 5.5 h (4.1–6.0), with a median drinking volume during the assessment of 1,400 mL (1,025–1,860) and median urine production of 660 mL (265–1,050). In 54 (55 %) patients, the evaluation led to permanent treatment with sacral neuromodulation. Twelve patients experienced faecal incontinence in addition to urgency urinary incontinence. In 67 % of these patients, faecal incontinence symptoms decreased by ≥50 % during SNM.

### Conventional-UDS and ambulatory-UDS outcomes compared

**Fig. 1:**
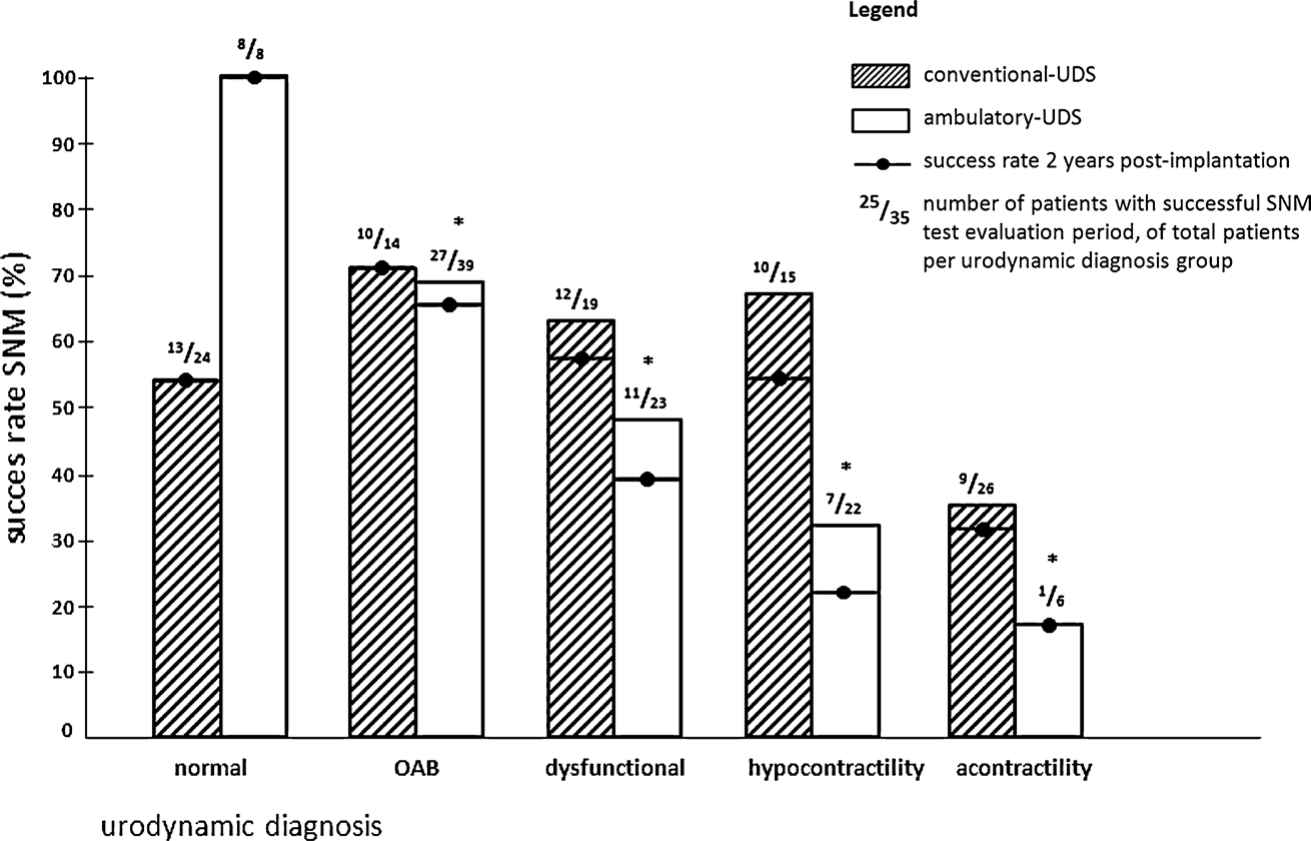
Storage and voiding related urodynamic diagnoses are not clustered by (extensive) symptom history. Success rate at long term (2 years) is additionally depicted. Normal: patients with subjective complaints, only represented in voiding diaries but not on conventional-UDS or ambulatory-UDS. Dysfunctional: patients with dysfunctional voiding or functional obstruction. Among the patients with hypocontractility there are also patients with filling phase contractions combined with impaired contractility during micturition phase. * Chance of success is significantly (*p* < 0.05) lower for the different ambulatory-UDS outcomes compared to a normal outcome. For conventional-UDS outcomes this is not the case (logistic regression analysis). *UDS* urodynamic study, *OAB* overactive bladder syndrome, *SNM* sacral neuromodulation

Overall there is a high association between conventional-UDS and ambulatory-UDS outcomes (Cramer’s *V* = 0.336 (*p* < 0.001), Pearson’s contingency coefficient: *C* = 0.558 (*p* < 0.001)) see Table 1, subset A. Success rates based on conventional-UDS are not significantly (*V* = 0.272 (*p* = 0.124), *C* = 0.262 (*p* = 0.124)) different from the success of patients with normal conventional-UDS recordings, no matter what the specific diagnosis, as can be seen in Fig. 1. The association between outcomes of conventional-UDS and successful SNM treatment is also not significant when stratified for patients based on history of storage or voiding dysfunction. This is in contrast to the relationship between outcomes of ambulatory-UDS and successful SNM treatment, as the there is a moderate association [*V* = 0.435 (*p* = 0.001), *C* = 0.399 (*p* = 0.001)], between them.

**Table 1:** Urodynamic confirmation between conventional and ambulatory urodynamic studies

Conventional-UDS outcome	Ambulatory-UDS outcome					Total
	Normal	OAB	Dysfunctional	Hypocontractile	Acontractile	
Subset A: 5 × 5 contingency table for conventional-UDS and ambulatory-UDS						
Normal	2	19	1	1	1	24
OAB	1	8	3	2	0	14
Dysfunctional	0	6	6	7	0	19
Hypocontractile	3	3	5	4	0	15
Acontractile	2	3	8	8	5	26
Total	8	39	23	22	6	98

Conventional-UDS diagnosis	Patients (*n*), SNM successful (%)	Confirmed on a-UDS (*n*), SNM successful (%)	SNM successful (*n*) in unconfirmed group (%)	Diagnosis solely on a-UDS (*n*) and success (%)
Subset B: patients clustered by history of storage symptoms				
OAB^a^	11 (64)	6 (83)	2 (40)	26 (62)
OAB with DO	7 (57)	4 (75)	1 (33)	21 (62)
Normal	18 (56)			
Voiding dysfunction	10 (60)			
Total	39 (59)^b^			

Conventional-UDS diagnosis	Patients (*n*), SNM failure (%)	Confirmed on a-UDS (*n*), SNM failure (%)	SNM failure in unconfirmed group (%)	Diagnosis solely on a-UDS (*n*) and failure (%)
Subset C: patients clustered by history of voiding symptoms				
Acontractile	23 (65)	5 (100)	10 (66)	1 (100)
Hypocontractile	9 (33)	4 (75)	0 (0)	18 (78)^c^
Dysfunctional voiding	18 (39)	8 (37)	4 (40)	10 (50)
Normal	6 (50)			
Storage dysfunction	3 (0)			
Total	59 (47)			

Cramer’s *V* = 0.336 (*p* < 0.001). Pearson’s contingency coefficient: *C* = 0.558 (*p* < 0.001)

When SNM is successful in patients with a history of storage dysfunction, there is a significant association between the conventional-UDS-based and the ambulatory-UDS-based diagnoses (*C* = 0.767, *p* < 0.001). If SNM is not successful, no relation is seen

*a-UDS* ambulatory urodynamic study, *c-UDS* conventional urodynamic study, *OAB* overactive bladder, *DO* detrusor overactivity

^a^OAB consists out of OAB-dry and OAB-wet

^b^The seven patients with OAB with DO are amongst the 11 OAB patients

^c^Of the 18 patients of hypocontractile on ambulatory-UDS, ten were only hypocontractile and the eight others had also filling phase contractions. In patients with a history of voiding dysfunction, no significant association is seen, independent of successful SNM

### Storage dysfunction

SNM treatment success rates were around 70 % in patients with a diagnosis of OAB after history taking and performance of urodynamics (Fig. 1). The total group was also stratified purely on history taking between (mainly) storage or voiding dysfunction. Of the 39 patients with a history of storage dysfunction, only 11 showed signs of OAB on conventional-UDS. On the other hand, 26 showed indications of OAB on ambulatory-UDS. Of these 39, only seven patients (18 %) showed DO on conventional urodynamics. On ambulatory-UDS of the same patients, 32 (82 %) showed contractions during the filling phase. These 32 patients included the seven patients with DO on conventional-UDS. If storage dysfunction was confirmed on both urodynamic tests, success rate was almost 40 % higher (Table 1, subset B) than when only seen on one of both tests (success rates were on average 79 %). If storage dysfunction was only seen on one of the two, success rate on average was 57 %, regardless of the urodynamic test. The presence or absence of DO or ‘detrusor contractions during the filling phase’ in addition to the storage symptoms did not change this similarity in success rate for both conventional-UDS and ambulatory-UDS. This is in concordance with the finding that when SNM is successful in patients with a history of storage dysfunction, there is a significant association between the conventional-UDS-based and the ambulatory-UDS-based diagnoses (*C* = 0.767, *p* < 0.001).

### Voiding dysfunction

Of the group of patients with a history of voiding dysfunction, 53 % (31 of 59) had a successful SNM evaluation period (Table 1, subset C). Of these 59 patients with a history of bladder emptying problems, 23 (39 %) were acontractile on conventional-UDS. On ambulatory-UDS, only 6 (10 %) of the 59 appeared to be acontractile. Hence, in 78 % of patients (18/23), the diagnosis of acontractility on conventional-UDS was not confirmed on ambulatory-UDS. All five patients with acontractility on both conventional-UDS and ambulatory-UDS failed the SNM test period. Eight (35 %) of these 23 patients showed hypocontractility on ambulatory-UDS, and 63 % of these patients underwent successful SNM test period. Additionally, nine patients showed hypocontractility on conventional-UDS, of which four were also hypocontractile on ambulatory-UDS. If hypocontractility was confirmed by ambulatory-UDS, failure rate was 40 % higher than when diagnosis was only based on conventional-UDS. Finally, three patients with a history of storage dysfunction appeared to be acontractile on conventional-UDS, and none of these patients were acontractile on ambulatory-UDS. These patients also had a successful neuromodulation test.

### Long-term response

Of all 98 patients evaluated, 54 (55 %) underwent permanent implantation as a result of significant reduction in symptoms during the test stimulation. After 1 year, 98 % still were successfully treated (without a decline of the initial improvement), after 2 years this percentage dropped to 94 %, as is shown in Fig. 1. Several patients (*n* = 19) are treated with SNM for a period longer than 5 years, and in this group most patients (89 %) still have a favourable effect of the treatment. In case patients show an effect during initial SNM evaluation, there is no significant difference (*p* > 0.05) between the different SNM indications with regard to long-term effect.

## Discussion

In the past, it has been attempted to identify which patients are more likely to benefit from treatment with SNM. It appears to be difficult to define reliable predictive factors [15, 16]. It is assumed that patients with a real acontractile bladder have a lower SNM treatment success rate [8]. However, how to determine this real acontractile bladder remained unclear [17]. Some also assumed that OAB patients with DO on urodynamics could have a higher potential treatment success, compared to the OAB patients without DO [15].

This study is relating urodynamic recordings, taking symptom presentation into account, to treatment outcome in SNM and could be a first step towards validation of the ambulatory-UDS. It provides clues that performing ambulatory urodynamics in selected patients will lead to a better patient selection and thus more successful SNM outcomes. In this study treatment, success rate amongst patients with storage dysfunction (around 70 %) was higher than in the patients with voiding dysfunction (around 50 %). The lowest success rate was seen in the patients with hypocontractility or acontractility on ambulatory-UDS (respectively, 32 and 17 %). In 78 %, the diagnosis of acontractility on conventional-UDS was not confirmed with ambulatory-UDS.

Although ambulatory-UDS is not validated yet, the recording of a detrusor contraction can easily be detected. An acontractile bladder confirmed on ambulatory-UDS is therefore definitely more reliable to be a real acontractile bladder.

Filling phase contractions were more abundant on ambulatory-UDS recordings than on conventional-UDS in this study. Both on conventional-UDS and on ambulatory-UDS, the presence of OAB with DO or filling phase contractions did not lead to a difference in outcome, compared to patients with OAB without these contractions. In another study, OAB patients were treated with OnabotulinumtoxinA bladder injections, and treatment outcome does not appear to be related to the pretreatment urodynamic finding of DO neither [18].

In general not only in symptomatic patients [6], but also in asymptomatic individuals, an increase in the number of detrusor contractions during the storage phase on ambulatory-UDS has been found [2, 19, 20]. It has been noted that about 50 % of patients are continuously aware of the catheter and have increased urge sensation, but they voluntarily suppress their reaction [2]. Detrusor overactivity has also been found on ambulatory-UDS in patients with only stress urinary incontinence symptoms [21, 22]. The significance of detrusor contractions without subsequent leakage in asymptomatic patients is unknown. The increased number of contractions can be elicited by catheter irritation or due to artifacts. It cannot be excluded nor proven that the bladder catheter is a non-physiological trigger and may explain the high incidence of detrusor overactivity [23]. The meaning of this higher false-negative rate in diagnosing DO on conventional-UDS compared to ambulatory-UDS has yet to be elucidated [24]. DO seen on ambulatory-UDS may have other diagnostic and therapeutic consequences than DO seen on conventional-UDS. Therefore, the meaning of DO on ambulatory-UDS needs further explanation. However, these findings will not change the conclusions based on our results, as was shown that the detection of DO does not predict a different SNM treatment outcome.

The patients included in this study are a clinically preselected group with more complex pathology, compared to other patients that did not undergo additional ambulatory-UDS. This can also explain the lower average success rate of SNM evaluation (54 %), as this is around 70 % in the total treatment group in our tertiary referral population. This patient selection raises a risk of bias, as ambulatory urodynamics is performed on specific indication, rather than randomly.

Another point of discussion is the representativeness of the diagnosis in repeated urodynamic studies. Regardless of the urodynamic study being ambulatory or conventional, the fact of having multiple urodynamic studies could lead to a higher recording rate of symptom related events. As most patients already underwent repeated conventional-UDS before additional ambulatory-UDS, the influence of this possible source of bias seems negligible.

Ambulatory urodynamic studies are more time-consuming than conventional-UDS. It remains debatable when they can be of additional value in clinical diagnosis and treatment evaluation. This additional evaluation should be reserved for patients in which conventional pressure flow studies have failed to fully explain or reproduce the symptoms and where further knowledge is likely to aid in subsequent management. Based on results from this study, we advise to perform ambulatory-UDS in patients with alleged diminished bladder contractility. The value of contractions during the filling phase with ambulatory-UDS in patients with OAB complaints, without DO on conventional-UDS, should be the subject of future research. Future studies should also define normal ranges for filling and voiding cystometry in ambulatory-UDS. This, in combination with the process of relating urodynamic results to treatment outcomes in patients with lower urinary tract symptoms, will be useful in the clinical validation of ambulatory-UDS.

## Conclusions

In patients with storage dysfunction, regardless of the presence of detrusor overactivity, additional ambulatory-UDS does not seem to contribute to a better prediction of success with SNM. However, it could be of importance in other treatments. Ambulatory-UDS is more sensitive in detecting detrusor contractions in patients with voiding dysfunction. The success rate amongst the patients with hypocontractility or acontractility is higher based on conventional-UDS than on ambulatory-UDS. The most likely cause of this discrepancy is the overestimation of the amount of patients with impaired contractility when relying on conventional-UDS. The diagnosis of a real acontractile bladder implicates an obvious high risk of failure of SNM. This information makes more accurate patient selection for SNM possible.

Therefore, ambulatory urodynamics could better guide treatment choice than conventional-UDS in these patients.

## Abbreviations

Ambulatory-UDS: Ambulatory urodynamic study
Conventional-UDS: Conventional urodynamic study
DO: Detrusor overactivity
DU: Detrusor underactivity
NOR: Non-obstructive urinary retention
OAB: Overactive bladder
SNM: Sacral neuromodulation
